# Supplementary figures and images for: Disclosing disabilities: Barriers for medical school applicants
Source: PLoS One. 2025 Aug 5;20(8):e0326880. doi: 10.1371/journal.pone.0326880 (PMC12324086; doi:10.1371/journal.pone.0326880)

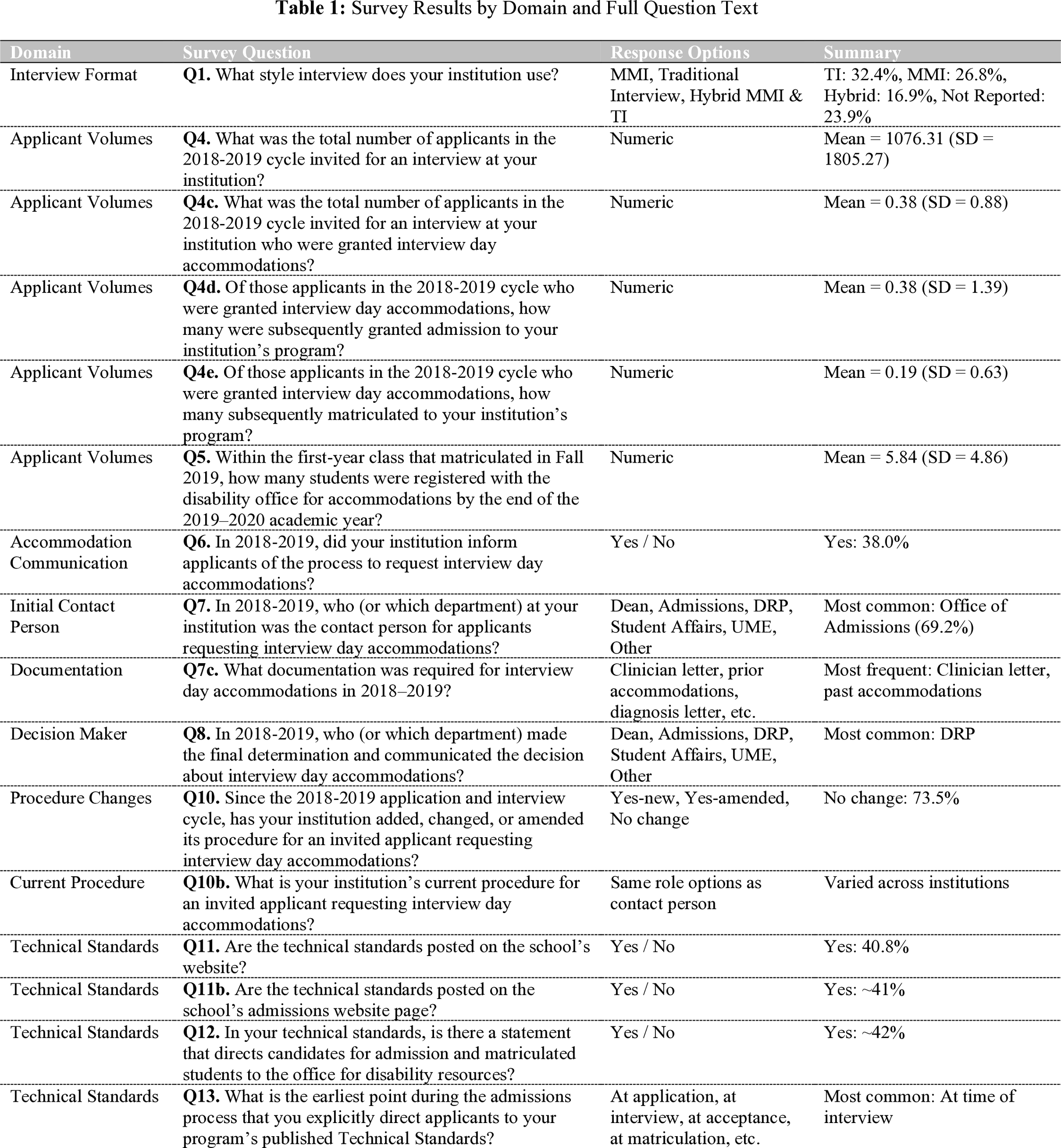

Supplement: S1 Table — Summary of institutional responses by domain, detailing interview formats, applicant volumes, accommodation processes, and technical standards. (TIF) [file pone.0326880.s001.tif]
