# Supplementary material for: Disclosing disabilities: Barriers for medical school applicants
Source: PLoS One. 2025 Aug 5;20(8):e0326880. doi: 10.1371/journal.pone.0326880 (PMC12324086; doi:10.1371/journal.pone.0326880)
Supplement: S1 Appendix — Instructions provided to survey respondents outlining the purpose, scope, reference year (2018–2019 cycle), and support resources for survey completion, including IRB exemption details. (DOCX) [file pone.0326880.s002.docx]

**S1 Appendix: Survey Instructions**

Thank you for participating in our research study.

When answering the following questions, please use your institution’s data and procedures from the **2018-2019 application and interview cycle for the class that matriculated in Fall 2019**.  This academic year was selected to avoid anomalies due to COVID-19.

We understand that as policies and practices evolve, your institution may have updated its procedures. You will have a chance at the end of this survey to comment on any recent updates.

Please note that a PDF copy of this survey has been emailed to your institution's Disability Resource Provider (DRP) to support you in completing the survey as there may be some questions that may necessitate their assistance or input.

If you have any questions or difficulties completing the survey, please email Dr. Kristina Petersen: k_harrispetersen@nymc.edu.

Note: This study has been classified as "Exempt" by the New York Medical College IRB. Click here to view the exemption.
