# Supplementary material for: Disclosing disabilities: Barriers for medical school applicants
Source: PLoS One. 2025 Aug 5;20(8):e0326880. doi: 10.1371/journal.pone.0326880 (PMC12324086; doi:10.1371/journal.pone.0326880)
Supplement: S2 Appendix — Full text of the 2021 survey distributed to medical school admissions deans and disability resource providers, covering 2018–2019 admissions data, interview day accommodation procedures, current practices, and technical standards. (DOCX) [file pone.0326880.s003.docx]

**S2 Appendix.** Survey Questions

**Section 1: Your Institution’s 2018-2019 Cycle Data**

*In this section, please provide numeric data about your institution’s 2018-2019 application and interview cycle. Please be as accurate as possible with the reported data. It may be helpful to consult with other departments (e.g. Student Affairs, Disabilities Resources, Academic Support, etc.) as needed.*

1. What was the total number of applicants in the 2018-2019 cycle **invited for an interview** at your institution?

1b. What style interview does your institution use?

- Multiple Mini Interview (MMI)
- Traditional Interview (TI)
- Hybrid MMI & TI

2. What was the total number of applicants in the 2018-2019 cycle who were subsequently **granted admission** to your institution’s program?

3. What was the size of the school of medicine's (SOM) entering class in Fall 2019?

4. What was the total number of applicants in the 2018-2019 cycle invited for an interview at your institution who **requested disability-related interview day accommodations**?

- My institution's data is (fill in): __________
- I do not know.

4b. Of the applicants invited for an interview who **requested disability-related interview day accommodations**, how many fit into the following categories? Note that this may require consultation with your institution's DRP or representative*.*

- Attention-deficit/hyperactivity disorder __________
- Learning disability __________
- Psychological disability __________
- Chronic health disabilities __________
- Mobility disability __________
- Visual disabilities __________
- Deaf or hard of hearing __________
- Other functional impairment __________
- I do not know __________

4c. What was the total number of applicants in the 2018-2019 cycle invited for an interview at your institution who were **granted interview day accommodations**?

- My institution's data is (fill in): __________
- I do not know.

4d. Of those applicants in the 2018-2019 cycle who were **granted interview day accommodations**, how many **were subsequently granted admission** to your institution’s program?

- My institution's data is (fill in): __________
- I do not know.

4e. Of those applicants in the 2018-2019 cycle who were **granted interview day accommodations**, how many **subsequently matriculated** to your institution’s program?

- My institution's data is (fill in): __________
- I do not know.

5. Within the first-year class that matriculated in Fall 2019, how many students were **registered with the disability office for accommodations** by the **end of the 2019-2020** academic year?

________________________________________________________________

**Section 2: Your Institution’s 2018-2019 Interview Day Accommodations Procedure**

*In this section, please consider your institution’s 2018-2019 interview day accommodations procedure. You will have an opportunity in the following section to describe your institution’s current procedures.*

6. In 2018-2019, when a student was invited for an admissions interview, did your institution inform students of a specific procedure for requesting accommodations for the interview day?

- Yes
- No

7. In 2018-2019, who (or which department) at your institution was the contact person for applicants requesting interview day accommodations?

- Dean of Admissions
- Office of Admissions
- Institution’s designated Disability Resource Provider (DRP)
- Office of Student Affairs
- Office of Undergraduate Medical Education
- Other (please describe): __________

7b. In 2018-2019, please specify what types of documentation applicants were required to provide to support their need for interview day accommodations. **Please select all that apply**.

- An application form that is **unique** for interview day accommodations.
- The **same** application used to determine student accommodations within your institution.
- At least one (1) letter from a clinician.
- Proof of past accommodations (e.g. undergraduate, standardized testing).
- No application form and/or documentation required.
- Other (please specify): ___________________________________________

7c. In 2018-2019, who made the ultimate determination about which accommodation requests were reasonable and communicated the accommodation plan to the applicant prior to interview day?

- Dean of Admissions
- Office of Admission staff member
- Institution’s Disability Resource Provider (DRP)
- Office of Student Affairs
- Office of Undergraduate Medical Education
- Other (please describe): __________________________________________

8. Where is the procedure published for applicants requesting accommodations for interview day within your institution’s policies and/or communication materials? (e.g. website, FAQ page, technical standards, direct communications with applicants)

________________________________________________________________

**Section 3: Your Institution’s Current Interview Day Accommodations Procedure**

*In this section, please consider your institution’s current interview day accommodations procedure.*

9. At your institution, to whom does the disability resource office **currently** report?

- Standalone office
- Office of Undergraduate Medical Education
- Office of Student Affairs
- Office of Diversity and Inclusion (University-Wide)
- Office of Diversity and Inclusion (only for School of Medicine)
- Other (please describe): _____________________________________________

10. Since the 2018-2019 application and interview cycle, has your institution added, changed, or amended its procedure for an invited applicant requesting interview day accommodations?

- Yes, the institution has established a **new** procedure.
- Yes, the institution has **amended** its previously established procedure.
- There has been **no change** since the 2018-2019 cycle.

10b. What is your institution’s **current procedure** for an invited applicant requesting interview day accommodations?

- Contact the Dean of Admissions
- Contact another staff member in the Office of Admission
- Contact the institution’s designated Disability Resource Provider (DRP)
- Contact the Office of Student Affairs
- Contact the Office of Undergraduate Medical Education
- Other (please describe): ______________________________________________

**Section 4: Your Institution’s Technical Standards (TS)**

*In this section, please consider your institution’s Technical Standards.*

11. Are the technical standards posted on the school’s website?

- Yes
- No

11b. Are the technical standards posted on the school’s **admissions** website page? O

- Yes
- No

11c. What is the **earliest point** during the admissions process that you explicitly direct applicants to your program’s published Technical Standards?

- Prior to submitting the supplemental application.
- When the applicant is invited for an interview.
- On the day of the applicant’s interview.
- After the applicant’s interview day but prior to a decision being rendered.
- After the applicant has been accepted to the program but prior to matriculation.
- After the applicant has matriculated to the program.
- Other (please describe): _____________________________________

12. In your technical standards, is there a statement that directs candidates for admission and matriculated students to the office for disability resources?

- Yes
- No

12b. Approximately when was the last date that your institution’s technical standards were revised? Please provide any rationale associated with these revisions.

___________________________________________________________________________________________________________________________________________________________________________
